# Long-term survival of a patient with small cell carcinoma of the stomach with metachronous lung metastases treated by multimodal therapy: a case report

**DOI:** 10.1186/s40792-015-0126-1

**Published:** 2015-12-24

**Authors:** Keishiro Aoyagi, Junya Kizaki, Taro Isobe, Yoshito Akagi

**Affiliations:** Department of Surgery, Kurume University School of Medicine, 67 Asahi-machi, Kurume, Fukuoka 830-0011 Japan

**Keywords:** Small cell carcinoma, Stomach, Multimodal therapy, Long survival, Lung metastasis

## Abstract

A 69-year-old man was referred to our institution for treatment of gastric cancer. Type 2 gastric cancer was found on the anterior wall of the lower body of the stomach.

The patient underwent distal gastrectomy, D2 lymph node dissection, and Roux-en-Y reconstruction with curative resection. The tumor was diagnosed as a small cell carcinoma of the stomach. Recurrence occurred in the lung after surgery. The patient underwent several chemoradiation therapy regimens, including cisplatin + irinotecan + radiation, S-1 + paclitaxel, amrubicin, carboplatin + etoposide, nogitecan, and docetaxel for lung metastases and radiation for brain and bone metastases for 43 months. He finally died of brain metastases 74 months after surgery (47 months after recognition of the lung metastases). Long continuous multimodal treatment including surgery, regimens for small cell lung cancer, S-1, taxanes, and radiation was thought to prolong the survival of this man with small cell carcinoma of the stomach.

## Background

The World Health Organization classification of 2010 defined neuroendocrine carcinoma (NEC) as a subgroup of neuroendocrine neoplasms. Neuroendocrine neoplasms are classified as neuroendocrine tumors or NECs according to their bioactivity, which is determined by the mitotic rate and Ki67 index [1]. The Japanese classification of gastric carcinoma defines NEC as a special type in the histological classification of gastric tumors and considers NEC to be either small cell type or large cell type [2]. NEC of the stomach is relatively rare, accounting for 0.1 to 0.6 % of all gastric carcinomas [3, 4]. A high frequency of capillary invasion and hematogenous metastases, such as to the liver and lung, is seen with NEC, and the prognosis is poor. Moreover, a standard chemotherapy regimen for NEC of the stomach has not yet been established.

We present a case involving a patient with small cell carcinoma of the stomach with metachronous lung metastases who survived for 74 months after surgery with multimodal therapy including surgery, chemotherapy, and radiation.

## Case presentation

**Fig. 1:**
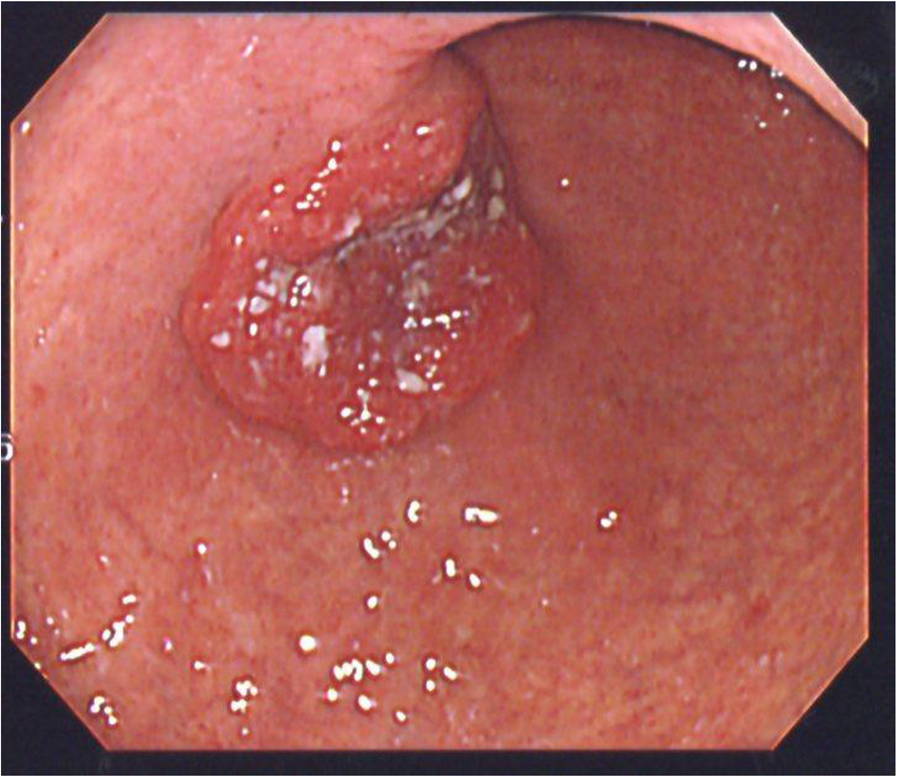
Upper gastrointestinal endoscopic examination. An irregular central ulceration with clearly demarcated and raised margins, type 2, is present on the anterior wall of the lower body of the stomach

A 69-year-old man with a history of diabetes mellitus and hypertension visited a local hospital with the complaint of a heavy feeling in the stomach and epigastric distress. An X-ray examination and upper gastrointestinal endoscopic examination revealed a type 2 tumor on the anterior wall of the lower body of the stomach. The pathological diagnosis based on a biopsy specimen from the tumor lesion was anaplastic carcinoma. The patient was referred to the Center of Gastroenterology, Kurume University Hospital, for further examination and treatment for gastric cancer on 7 December 2005. The liver, spleen, and tumor were not palpable on physical examination. The serum levels of carcinoembryonic antigen, carbohydrate antigen 19-9, and cancer antigen 72-4 were within the reference range. An X-ray examination showed an irregular round ulcerative lesion surrounding a well-demarcated, smooth protrusion on the anterior wall of the lower body of the stomach. An upper gastrointestinal endoscopic examination showed an irregular central ulceration with clearly demarcated and raised margins, type 2, on the anterior wall of the lower body of the stomach (Fig. 1). The pathological diagnosis based on a biopsy specimen was a poorly differentiated adenocarcinoma. Abdominal computed tomography (CT) showed no metastatic lesions in the liver and no lymph node metastases. The patient was admitted to the hospital and underwent distal gastrectomy with D2 lymph node dissection and Roux-en-Y reconstruction on 25 January 2006.

**Fig. 2:**
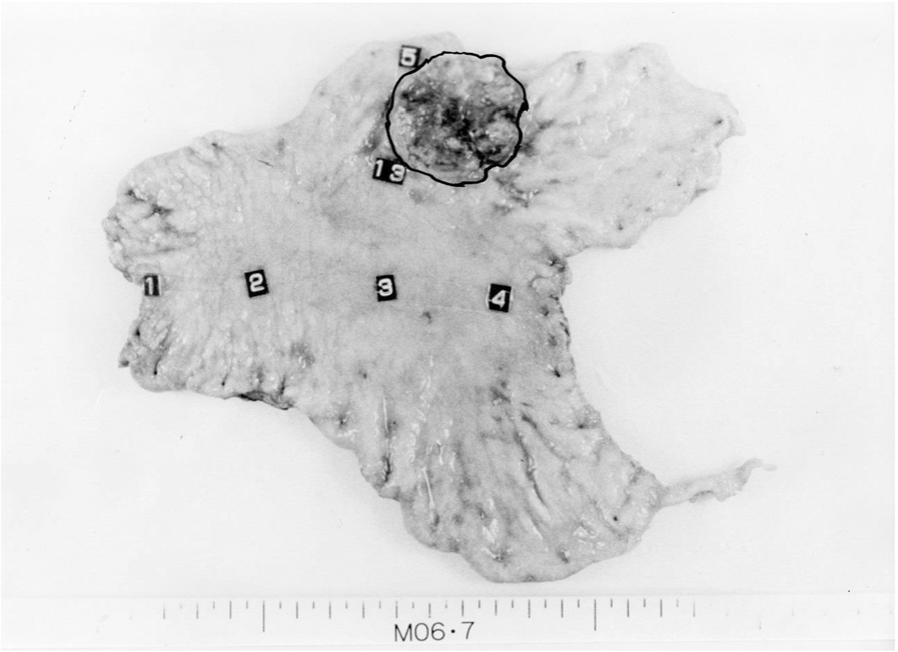
Macroscopic examination. The specimen resected from the anterior wall of the lower body was sharply demarcated, type 2, and 43 × 41 mm in size

**Fig. 3:**
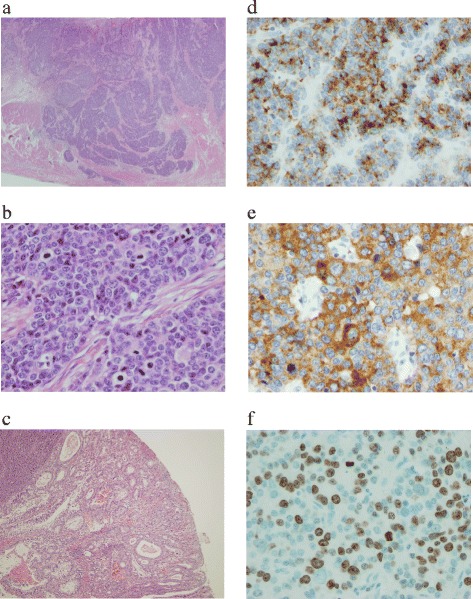
Hematoxylin-eosin staining and immunohistochemistry. **a** The tumor is invading the muscularis propria in some parts (HE staining, ×40). **b** Monotonous tumor cells have little cytoplasm and round chromatin-rich nuclei; many mitotic figures are also seen (183 per 10 high-power fields) (HE staining, ×400). **c** In one part of the surrounding elevation, a well-differentiated tubular adenocarcinoma is seen (HE staining, ×40). The tumor shows positive staining with **d** chromogranin A (×400) and **e** synaptophysin (×400). **f** Many Ki67-positive cells are seen, and the Ki67 index is 66.7 % (×400)

The primary tumor was palpable on the anterior wall of the lower body, and no exposed tumor was seen on the serosa. No macroscopic metastases were found in the lymph nodes, liver, or peritoneum. On macroscopic examination, the specimen resected from the anterior wall of the lower body was sharply demarcated, type 2, and 43 × 41 mm in size (Fig. 2). A lower-power view of a cross section of the specimen showed medullary infiltration of neoplastic cells, mainly in the submucosa. A low-power histological view showed that the tumor had invaded the muscularis propria in some parts (Fig. 3a). The high-power view showed monotonous tumor cells with little cytoplasm and round chromatin-rich nuclei; many mitotic figures were also seen (183 per 10 high-power fields) (Fig. 3b). A well-differentiated tubular adenocarcinoma was recognized in one part of the surrounding elevation (Fig. 3c). The tumor showed positive immunohistochemical staining with chromogranin A and synaptophysin (Fig. 3d, e). The Ki67 index was 66.7 % (Fig. 3f). Thus, the histological diagnosis was small cell carcinoma of the stomach with moderate lymphatic and venous invasion. No metastases were seen in the lymph nodes on histological examination. The final diagnosis was a gastric cancer type 2 (LM), T2N0H0P0M0 stage IB. The surgery was evaluated as a curative resection with a negative resection margin (R0). The patient’s postoperative course was good, and he was discharged from the hospital on 10 February 2006.

**Fig. 4:**
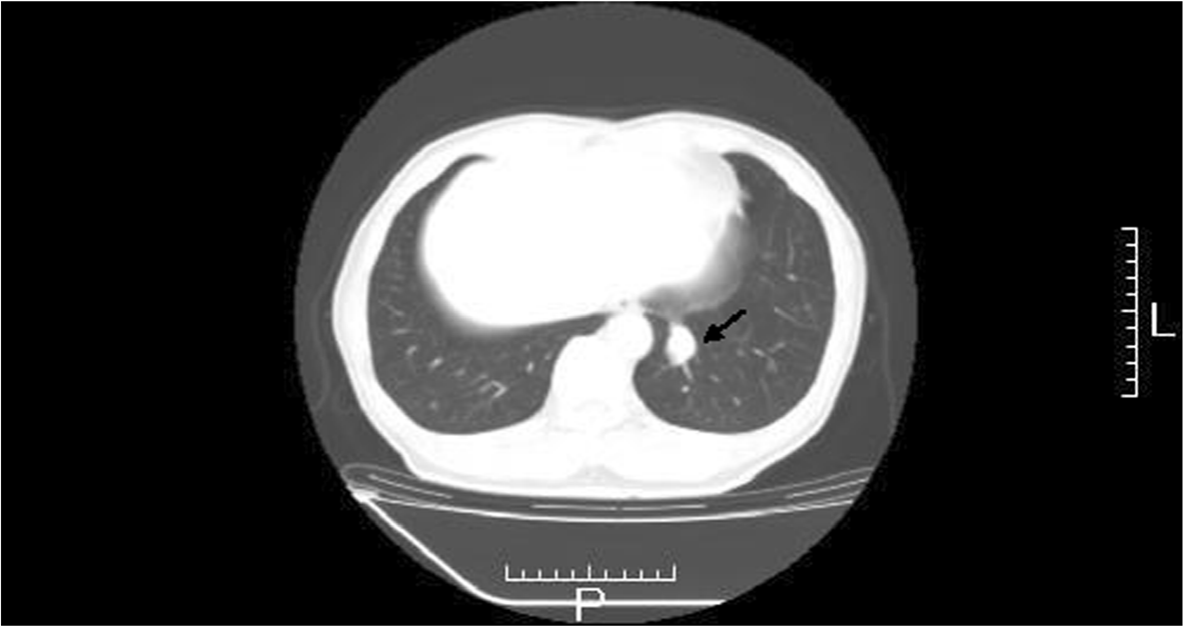
Chest computed tomography on 21 May 2008. Chest computed tomography shows a well-demarcated 20-mm mass at S10 of the left lung (*arrow*)

**Fig. 5:**
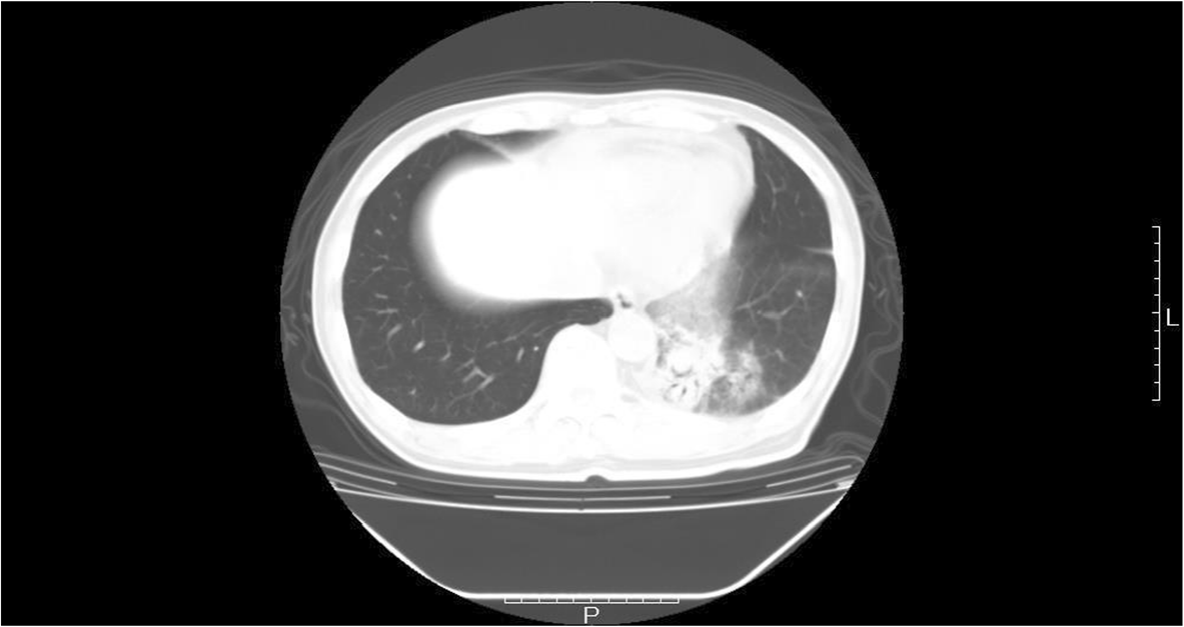
Chest computed tomography on 21 January 2009. The mass at S10 of the left lung is not detected

**Fig. 6:**
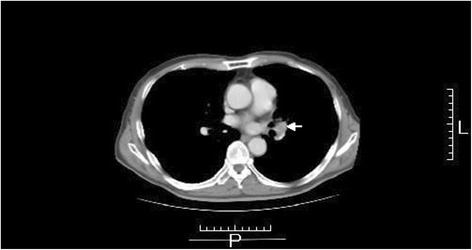
Chest computed tomography on 31 August 2010. Swelling of a lymph node, 20 mm in size, is recognized at the hilum of the left lung (*arrow*)

**Fig. 7:**
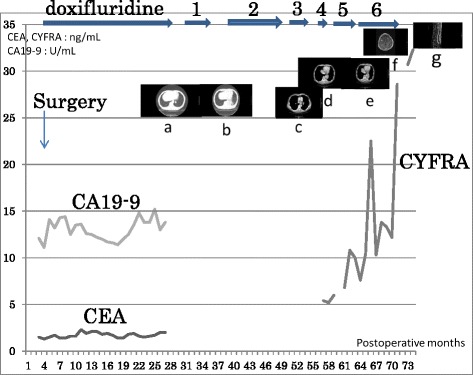
Summary of postoperative course. After recurrence in the lung at 27 months after surgery, the patient was administered first-line chemotherapy with radiation for 5 months. The clinical effect was CR. However, regrowth was recognized at 39 months. We administered second-line chemotherapy for 10 months and third-line chemotherapy for 4 months. The tumor slightly increased. Therefore, we administered fourth-line chemotherapy for 2 months. However, the patient had severe adverse effects. Moreover, the tumor increased and a swollen lymph node was recognized at 58 months. We changed to fifth-line chemotherapy. However, the tumor increased and effusion developed at 64 months. We administered sixth-line chemotherapy for 7 months. The tumor in the lung had not changed, but brain (69 months) and bone metastases (72 months) were recognized. The serum level of CYFRA was initially slightly elevated; it became upregulated with the development of progressive disease, finally reaching >30 ng/mL with brain and bone metastases. The CEA and CA19-9 levels were within the reference range

The patient was then administered 600 mg of doxifluridine as postoperative adjuvant chemotherapy for 27 months. However, a lung tumor was seen in the left lower lung field on the chest X-ray on 28 April 2008. Chest CT showed a well-demarcated mass, 20 mm in size, at S10 of the left lung (Fig. 4). Small cell carcinoma cells were confirmed by transbronchial lung biopsy. Thus, this tumor was diagnosed as lung recurrence of gastric small cell carcinoma. The patient began treatment with chemotherapy comprising cisplatin (CDDP) (90 mg, day 1) + irinotecan (CPT-11) (90 mg; days 1, 8, and 15) (28-day cycle); radiation of 45 Gy (150 cGy × 2 × 15 days) was added. After six courses of this regimen, chest CT revealed no mass at S10 of the left lung on 21 January 2009 (Fig. 5). The patient was then followed carefully without chemotherapy. Chest CT subsequently revealed regrowth of the mass at S10 of the left lung on 15 April 2009. The patient was administered S-1 (80 mg, days 1–14) + paclitaxel (PTX) (70 mg, days 1 and 8) (21-day cycle) as second-line chemotherapy from 12 May 2009 to 10 March 2010. Although nine courses of this regimen were administered, the size of the mass at S10 of the left lung increased. Amrubicin (60 mg, days 1–3) (21-day cycle) was then administered as third-line chemotherapy in six courses from 6 April to 11 August 2010. However, chest CT on 31 August 2010 showed that the mass lesion at S10 had increased to 9 × 26 mm and a lymph node at the hilum of the left lung had swollen to 20 mm (Fig. 6). Carboplatin (CBDCA) (280 mg, day 1) + etoposide (130 mg, days 1–3) (21-day cycle) was then administered in four courses as fourth-line chemotherapy from 28 September to 4 December 2010. The serum level of cytokeratin 19 fragment (CYFRA) was slightly elevated (Fig. 7) but that of neuron-specific γ-enolase (NSE) was within the reference range during fourth-line chemotherapy. Grade 3 and 4 adverse events were leukopenia and neutropenia, respectively. However, these adverse events were recovered during administration of a granulocyte colony-stimulating factor. Chest CT on 7 December 2010 showed that the mass lesion at S10 had increased to 46 × 30 mm and that the swollen lymph node at the hilum of the left lung had increased to 25 mm. Nogitecan (1.1 mg, days 1–5) (28-day cycle) was thus administered as fifth-line chemotherapy in six courses from 5 January to 21 May 2011. Chest CT then revealed that the mass lesion at S10 increased from 48 × 32 mm on 1 March to 65 × 40 mm on 7 June 2011 and a small amount of effusion had developed; however, the lymph node at the hilum of the left lung had not changed in size. Docetaxel (70 mg, day 1) (21-day cycle) was then given as sixth-line chemotherapy in eight courses from 14 June 2011 to 16 January 2012. Chest CT on 6 September 2011 revealed that the mass at S10 had not changed; however, the swollen lymph node at the hilum of the left lung had decreased from 30 × 26 mm to 26 × 18 mm.

Severe (grade 3 or 4) adverse effects did not occur except those associated with CBDCA + etoposide.

The patient developed bradypragia and higher brain dysfunction in mid-October 2011. Brain magnetic resonance imaging (MRI) revealed multiple brain metastases. Moreover, the patient developed lower back pain in mid-January, and the serum level of CYFRA increased to >30 ng/mL (Fig. 7). MRI of the thoracic and lumbar vertebrae on 31 January 2012 showed bone metastases in the thoracic vertebrae.

The patient was transferred to another hospital for palliative care. He died of brain metastases 74 months after surgery (47 months after recognition of the lung metastases).

### Discussion

For patients with gastric NEC, the National Comprehensive Cancer Network guideline recommends multimodal therapy including surgical resection of the stomach and postoperative administration of a regimen for small cell lung cancer, such as CDDP + etoposide (ETP), CDDP + CPT-11, or CBDCA + ETP [5]. In our case, the patient had been administered doxifluridine as postoperative adjuvant chemotherapy for 27 months; nevertheless, recurrence from gastric small cell carcinoma was recognized. Therefore, we concluded that doxifluridine had no clinical effect on this cancer and started new treatment comprising CDDP + CPT-11 + radiation. Various regimens for small cell lung cancer were administered in the present case, including CDDP + CPT-11 + radiation (first-line), amrubicin (third-line), CBDCA + ETP (fourth-line), and nogitecan (fifth-line) (Fig. 7). After first-line therapy, a complete response of the metastatic site in the lung was confirmed on chest CT. S-1 alone or S-1 + CDDP has been administered as the standard treatment for advanced gastric cancer based on the Japanese gastric cancer treatment guideline [6] and two phase-III trials (SPIRITS trial: S-1 vs. S-1 + CDDP [7] and JCOG 9912 trial: fluorouracil vs. CPT-11 + CDDP vs. S-1 [8]). Additionally, several case reports documented good clinical effects of S-1 or S-1 + CDDP for NEC of the stomach in Japan [9–11]. Hainsworth et al. [12] performed a phase-II trial of three anticancer drugs including taxane (PTX, CBDCA, and ETP) in patients with poorly differentiated NEC of the gastrointestinal tract and reported a response rate of 53 % and median progression-free survival of 14.5 months. In the present case, no severe adverse effects occurred with the administration of S-1 + PTX as second-line therapy (nine courses for 10 months) and docetaxel alone as sixth-line therapy (eight courses for 7 months) (Fig. 7). Some case reports have described good clinical responses with multiple combined chemotherapy for NEC of the stomach, such as CDDP + cyclophosphamide + epirubicin + vincristine sulfate, and ETP + CDDP + doxorubicin hydrochloride [13, 14]. However, the adverse effects of these regimens were severe. All six regimens administered in the present case involved one or two drugs, and it was possible to continue the chemoradiation therapy for 43 months (Fig. 7).

While the patient underwent the first regimen, the metastatic single nodule was localized to S10 of the left lung; thus, the radiation was thought to be effective as local therapy for this metastatic nodule. Moreover, radiation was performed for brain and bone metastasis and was thought to be useful for prolonged survival and improvement in the patient’s quality of life.

This case suggests that the addition of S-1 and taxane to a small cell lung cancer regimen with radiation prolongs the prognosis of recurrent NEC of the stomach.

NSE is reportedly a sensitive tumor marker for neuroendocrine tumors [15], but the NSE level was within the reference range in the present case. Although CYFRA is a sensitive marker for non-small-cell lung cancer [16], it was a very sensitive marker for progression and metastases in the present case (Fig. 7).

## Conclusions

The long-term continuous administration of regimens for small cell lung cancer, including S-1 and taxanes with one or two drugs and radiation, was thought to be effective against recurrence of small cell carcinoma of the stomach in the present case.

## Consent

Written informed consent was obtained from the patient’s wife for publication of this case report and any accompanying images. A copy of the written consent is available for review by the Editor-in-Chief of this Journal.

## Abbreviations

CBDCA: carboplatin
CDDP: cisplatin
CPT-11: irinotecan
CT: computed tomography
CYFRA: cytokeratin 19 fragment
ETP: etoposide
MRI: magnetic resonance imaging
NEC: neuroendocrine carcinoma
NSE: neuron-specific γ-enolase
PTX: paclitaxel
